# The effect of cariprazine on patient engagement: Post-hoc analysis of a phase 3 study in patients with predominant negative symptoms of schizophrenia

**DOI:** 10.1192/j.eurpsy.2021.1423

**Published:** 2021-08-13

**Authors:** I. Laszlovszky, Z.B. Dombi, Á. Balogh, Á. Barabássy, G. Vass, B. Szatmári, G. Németh

**Affiliations:** Medical Division, Gedeon Richter Plc., Budapest, Hungary

**Keywords:** Cariprazine, schizophrénia, patient engagement, negative symptoms

## Abstract

**Introduction:**

Motivation deficit is a significant aspect of lack of improvement in patients with schizophrenia especially with predominant negative symptoms (PNS). Therefore, improvement depends not only on symptoms reduction and better social functioning but also on patient engagement which is a key but less investigated aspect of successful treatment.

**Objectives:**

To investigate and compare patient engagement in PNS patients after cariprazine and risperidone treatment characterized by the 11 items of the Positive and Negative Syndrome Scale (PANSS-11).

**Methods:**

In this phase 3 study patients suffering from PNS of schizophrenia (PANSS-FSNS≥24) were randomized to 26 weeks of treatment with either cariprazine or risperidone (target dose 4.5 and 4 mg/day, respectively). To compare the effects of the two drugs on patient engagement the PANSS-11 scale was used. Change from baseline (CfB) on the selected items and PANSS-11 total score were analyzed using mixed model of repeated measures approach without correction for multiplicity.

**Results:**

PANSS-11 total score mean CfB were -11.20 (SD=0.43) for cariprazine-, and -9.44 (SD=0.45) for risperidone-treated patients with a -1.79 (95% CI=-3.01, -0.56) mean difference (p=0.004) in favor of cariprazine. Most item differences were statistically significant (N1, N2, N3, N4, N5, G16) or numerically higher (N6, G7, G13) for cariprazine versus risperidone.

**Conclusions:**

Cariprazine significantly improved patient engagement in patients with PNS of schizophrenia compared to risperidone based on the PANSS-11 post-hoc analysis. These results suggest that cariprazine treatment may improve not only the symptoms and everyday functioning of PNS patients but their engagement with life.

**Conflict of interest:**

Studies were funded by Gedeon Richter Plc. and Allergan Plc (prior to its acquisition by AbbVie). Dr. Laszlovszky, Dombi, Balogh, Dr Barabassy, Dr Vass, Dr. Szatmári and Dr. Németh are employees of Gedeon Richter Plc.

